# Synaptic Plasticity Fragility Underlies a Microglial Pruning Continuum in Major Depressive Disorder and Amyotrophic Lateral Sclerosis

**DOI:** 10.7759/cureus.107259

**Published:** 2026-04-17

**Authors:** Ngo Cheung

**Affiliations:** 1 Psychiatry, Cheung Ngo Medical Centre, Hong Kong, HKG

**Keywords:** als, depression, motoneuron diseases, neuronal plasticity and repair, plasticity

## Abstract

Background

Major depressive disorder (MDD) and amyotrophic lateral sclerosis (ALS) are clinically distinct yet show intriguing comorbidity, often early in the disease course. We hypothesized a shared microglia-mediated synaptic pruning vulnerability, amplified differently by disorder-specific pathways, autophagy collapse in ALS versus RNA processing and immune dysregulation in MDD, thereby creating a biological continuum.

Methods

Using large-scale genome-wide association study (GWAS) from the Psychiatric Genomics Consortium (PGC) (MDD, N=829,249) and Project MinE (ALS, effective N=87,381), we applied Multi-marker Analysis of GenoMic Annotation (MAGMA) for gene- and set-level associations, Gene Set Enrichment Analysis (GSEA)/Differential Gene Set Enrichment Analysis (DGSEA) for pathway enrichment and differential enrichment, S-PrediXcan transcriptome-wide association study (TWAS) across 14 GTEx tissues, and linkage disequilibrium score regression (LDSC) for partitioned heritability and cross-trait genetic correlation. Eight gene sets (housekeeping controls, monoaminergic, neurosteroid, glutamatergic, synaptic pruning, autophagy/protein quality, RNA processing, and immune/neuroinflammation) were tested for convergence and divergence.

Results

Synaptic pruning emerged as the sole consistent cross-disorder signal, with robust enrichment in MDD (LDSC 1.32×, GSEA NES=1.415, p=0.0001) and nominal but consistent signals in ALS (GSEA NES=1.40, p=0.011; TWAS HLA-B). Autophagy dominated ALS (LDSC 2.20×, TWAS C9orf72 Z=13.43, GSEA NES=1.94) but was depleted in MDD. RNA processing and immune pathways were prominent in MDD (LDSC 1.48× and 1.89×, respectively), with only nominal signals in ALS. Overall genetic correlation was near zero (rg=-0.044, p=0.196).

Conclusions

These findings support a microglial pruning continuum model: shared pruning liability as the foundation, with autophagy failure driving ALS neurodegeneration and RNA/immune dysregulation shaping MDD stress sensitivity. The low rg explains the modest overlap, while pathway specificity accounts for comorbidity and divergent progression. This framework offers testable predictions for polygenic risk score (PRS) stratification, complement modulators in ALS mood subsets, and microglial therapies in treatment-resistant MDD.

## Introduction

Clinical overlap between ALS and depression

Anyone who has spent enough time caring for people with amyotrophic lateral sclerosis (ALS) will eventually notice something hard to ignore: a good number of patients describe a flat, lingering sadness that seems to have been present well before the twitching or weakness ever started. The natural assumption is that the mood disturbance is a reaction to the diagnosis itself, and sometimes it is. But when the pattern keeps showing up, low mood preceding the first fasciculation by months, appearing too early and too consistently to be explained away as adjustment, it begins to feel like something more than coincidence [1,2]. Major depressive disorder (MDD) and ALS have traditionally been treated as fundamentally different problems, one rooted in psychiatry and the other in neurodegeneration. MDD disrupts mood, motivation, and cognition; ALS strips away voluntary movement through the relentless loss of upper and lower motor neurons, ending in respiratory failure [3,4]. Yet the epidemiological overlap is hard to dismiss. Systematic reviews and earlier clinical surveys place the prevalence of depression in ALS somewhere between 10% and 34%, with many cases emerging early enough in the disease course to raise the question of shared biological origins rather than simple psychological burden [1,2]. Once you start pulling on that thread, asking whether a common mechanism could connect motor neuron loss to emotional disturbance, the genetics become impossible to ignore.

Genetic architecture of ALS and MDD

Motor neuron diseases are a heterogeneous group, and ALS sits at the severe end of the spectrum. The core pathology involves progressive degeneration of both upper and lower motor neurons, producing muscle weakness, spasticity, and ultimately respiratory compromise [3]. Most cases arise sporadically, but roughly 10% to 15% have a clear genetic component, and large-scale sequencing has now implicated well over 100 genes [3,4]. In populations of European descent, the hexanucleotide repeat expansion in C9orf72 accounts for the largest share of familial cases, though mutations in SOD1, TARDBP, and FUS each contribute through partly distinct but converging pathways, protein aggregation, impaired RNA handling, and breakdown of autophagy [5,6]. MDD, on the other hand, has no single heavyweight mutation. It is polygenic through and through, with genome-wide meta-analyses now pointing to more than 100 risk loci that cluster in prefrontal and limbic brain regions and implicate disrupted synaptic plasticity, immune signaling, and stress-responsive gene networks [7,8].

The gap in understanding shared biology

Given these different genetic landscapes, it is perhaps not surprising that formal estimates of genetic correlation between ALS and MDD have generally hovered close to zero, suggesting, at least on the surface, that the two conditions occupy separate biological territories [9]. Previous cross-trait genomic studies have examined polygenic overlap between ALS and neuropsychiatric disorders, most notably McLaughlin RL et al. [9], who identified a modest but significant genetic correlation between ALS and schizophrenia (rg = 14.3%). Cross-disorder psychiatric analyses, such as Gandal MJ et al. [10], have mapped shared molecular neuropathology among psychiatric conditions using transcriptomic approaches. However, no study to date has applied a multi-method, pathway-level framework, combining gene-set association, pathway enrichment, tissue-specific transcriptomic imputation, and partitioned heritability, to systematically compare ALS and MDD at the level of biologically defined gene sets. This leaves a critical gap: while the genome-wide genetic correlation between ALS and MDD is known to be near zero, the possibility of convergence at specific biological pathways remains untested.

One area of emerging interest is microglia-mediated synaptic pruning, a process that appears to be dysregulated in both disorders, though in different ways [10,11]. In ALS, excessive or misdirected pruning tends to coincide with collapse of autophagy, fueling the toxic protein accumulation that characterizes the disease. In MDD, pruning abnormalities seem to intersect more with RNA processing disturbances and chronic, low-grade immune activation that keeps neural circuits under sustained stress [12,13]. Thinking of this as a kind of “pruning continuum” is not meant to be a grand unifying explanation. It is better described as a working framework, one that tries to account for why some ALS patients develop mood changes early in the disease, and why depression in the context of neurodegeneration often feels biologically rooted rather than purely reactive.

Study aims and analytical approach

We designed this study to test that framework using a layered set of genomic methods: MAGMA for gene-level and gene-set associations [14], Gene Set Enrichment Analysis (GSEA) and Differential Gene Set Enrichment Analysis (DGSEA) for pathway enrichment and cross-disorder differences [15], TWAS to connect genetic variants with tissue-specific gene expression [16], and linkage disequilibrium score regression (LDSC) for partitioned heritability and cross-trait genetic correlation [17,18]. Our analyses centered on eight biological gene sets, housekeeping controls, monoaminergic systems, neurosteroids, glutamatergic signaling, synaptic pruning, autophagy and protein quality control, RNA processing, and immune/neuroinflammation pathways. The intention was not to prove any single mechanism, but to map the points at which these two disorders converge and where they part ways. The hypothesis we sought to evaluate was whether shared pruning dysfunction acts as a quiet biological foundation, with disorder-specific amplifiers, autophagy failure tipping the balance toward ALS, and immune and RNA dysregulation toward MDD, shaping which clinical picture ultimately emerges.

Specifically, the objectives of this study were: (1) to determine whether microglia-mediated synaptic pruning gene sets are enriched in both ALS and MDD genome-wide association study (GWAS) signals, consistent with a shared biological substrate; (2) to test whether autophagy/protein quality control pathways show ALS-specific enrichment and whether RNA processing and immune pathways show MDD-specific enrichment, consistent with disorder-specific amplifiers; and (3) to estimate the genome-wide genetic correlation between ALS and MDD and reconcile it with any pathway-level convergence or divergence observed. To our knowledge, this is the first study to apply MAGMA, GSEA/DGSEA, TWAS, and partitioned LDSC in combination to compare ALS and MDD across biologically defined gene sets, thereby providing a novel pathway-resolution perspective on the comorbidity between a neurodegenerative and a neuropsychiatric condition.

What follows is our effort to make sense of the data, offered not as a definitive answer but as a scaffold for thinking about ALS and MDD as related points on a biological spectrum rather than as isolated conditions. If even a portion of this framework proves durable, it could have practical consequences for how we screen for mood disturbance in motor neuron disease, how we counsel patients who sit at the intersection of motor loss and emotional fragility, and how we think about treatment.

## Materials and methods

MAGMA gene-based and gene-set analysis

Gene-based association testing was carried out with MAGMA v1.10 [14], drawing on ALS GWAS summary statistics released by the Project MinE consortium [6]. That dataset included 27,205 cases and 110,881 controls; after harmonization, the effective sample size came to 87,381. We mapped single-nucleotide polymorphisms (SNPs) to genes using a window of 35 kb upstream and 10 kb downstream of each gene, following NCBI 37.3 coordinates, which left us with 18,222 protein-coding genes in the analysis. For each gene, a p-value was obtained from the mean chi-squared statistic of its constituent SNPs, with linkage disequilibrium structure accounted for through the 1000 Genomes European reference panel [19]. For the gene-set stage, we assembled eight biological pathways informed by the motor neuron disease literature [3,4]. These gene sets were manually curated by the investigator based on published reviews of ALS and MDD pathobiology [3-8,10,11], supplemented by genes drawn from established databases, including Gene Ontology (GO), Kyoto Encyclopedia of Genes and Genomes (KEGG), and Reactome, where available. The eight sets comprised: (1) housekeeping controls (ribosomal, metabolic, and cytoskeletal maintenance genes); (2) monoaminergic signaling (serotonin, dopamine, and norepinephrine pathway genes); (3) neurosteroid metabolism; (4) glutamatergic neurotransmission; (5) synaptic pruning (complement cascade, MHC class I, microglial receptors, and cell-adhesion molecules); (6) autophagy and protein quality control (autophagosome-lysosome pathway, ubiquitin-proteasome system, and chaperone-mediated clearance genes); (7) RNA processing (splicing, RNA transport, and RNA-binding protein genes); and (8) immune and neuroinflammation (toll-like receptors, cytokine signaling, and NF-κB pathway genes). Full gene lists for each set, including source annotations and database cross-references, are provided in Appendix 1. After removing genes that appeared in more than one set, always giving priority to the set higher in our predetermined hierarchy, so that housekeeping genes, for instance, were removed from the immune set rather than the other way around, we retained 851 unique genes across the eight pathways. Enrichment was evaluated with competitive t-tests comparing the average Z-statistic of genes within each set against the genome-wide background, and significance was judged after Bonferroni correction across the eight sets (threshold, 0.00625). The entire workflow ran through a custom pipeline built to handle both the PGC MDD 2025 TSV format and GWAS-VCF files, so that both disorders could be processed in a consistent manner.

GSEA and differential GSEA (DGSEA)

To examine pathway-level enrichment from a different angle than the MAGMA competitive tests, we ran gene-set enrichment analysis following the approach of Subramanian A et al. [15], using the MAGMA-derived Z-statistics as the ranking metric. For finer resolution, the eight core pathways were broken out into 115 subsets, though the original eight were kept intact for the differential comparisons. Each set was tested with 10,000 permutations, weighted enrichment scoring, and size filters requiring at least 5 and no more than 500 genes. We considered a set significant if it passed a false discovery rate threshold of 0.05 within each disorder separately. We then moved to differential GSEA, modeled on the cross-disorder framework described by Gandal MJ et al. [10]. This was done exhaustively across every pairwise combination of the eight major sets between ALS and MDD, again with 10,000 permutations, so that we could directly ask whether a given pathway was more enriched in one disorder than in the other. To support comparison across the two GWAS samples, gene sets were aligned and the results organized into pivot tables reporting normalized enrichment scores and p-values for the major pathways. The whole procedure was run through a custom Python pipeline that read MAGMA outputs and saved full results, including leading-edge gene lists, as CSV files.

TWAS using S-PrediXcan

We used S-PrediXcan [16] together with GTEx v8 MASHR prediction models [20] to connect GWAS signals to tissue-specific gene expression. The ALS analysis relied on the Project MinE summary statistics (effective N = 87,381), while the MDD analysis used the PGC 2025 meta-analysis (N = 829,249) [8]. Both sets of summary statistics were brought into a common format containing SNP identifier, chromosome, base-pair position, effect allele and other allele, beta, standard error, p-value, and sample size. For GWAS files distributed in VCF style, effect sizes and standard errors were extracted directly from the FORMAT fields (ES:SE:LP); PGC TSV files were handled by parsing effective sample size and case-control counts. Prediction models covered 14 tissues, and we gave priority to brain regions, frontal cortex (Brodmann area 9), hippocampus, and spinal cord, alongside whole blood. Gene-level Z-scores were calculated as weighted sums of SNP-level Z-scores divided by the square root of the prediction variance obtained from the covariance matrix. Enrichment of the eight gene sets was then tested with Mann-Whitney U tests comparing absolute Z-scores between target genes and the remaining background, corrected for multiple testing by tissue using the false discovery rate. We summarized the results across all tissues, identified the best-performing tissue for each gene, and carried out set-level comparisons. A Python pipeline managed the full workflow and handled the different GWAS input formats automatically.

LD score regression for partitioned heritability

To ask whether our eight gene sets captured a disproportionate share of common-variant heritability, we turned to partitioned LD score regression [18]. LD scores were computed from the European subset of the 1000 Genomes Project [19], and we used one-tailed tests oriented toward positive enrichment. Summary statistics were converted to the format expected by LDSC through a munging step, with a manual fallback procedure for VCF- and PGC-formatted files. For each of the eight pathways, we built annotation files by padding gene boundaries by 10 kb on each side and writing BED files, then computed LD score annotations across all 22 autosomes. The partitioned heritability analysis was run with the overlap-annotation flag enabled. Enrichment was expressed as the ratio of the observed proportion of heritability in a given annotation to the proportion expected from SNP coverage alone, and standard errors came from jackknife resampling. In cases where the full partitioned LDSC pipeline could not converge, typically when an annotation covered a very small fraction of total SNPs, resulting in unstable coefficient estimates, we applied a simplified fallback procedure. This involved stratifying SNPs by their annotation membership and computing the ratio of mean chi-squared statistics for annotated versus unannotated SNPs, adjusted for differences in local LD estimated from the 1000 Genomes European panel. This approach yields a conservative approximation of heritability enrichment and has been used in analogous contexts where annotation-specific LD score computation is numerically unstable because of sparse coverage [17,18]. We note that this fallback was invoked only for the smallest gene sets (autophagy and RNA processing in certain configurations) and that results from the full and simplified procedures were concordant in direction and magnitude where both could be computed. Results for both disorders were corrected across the eight sets using both Bonferroni and false discovery rate methods.

LD score regression for genetic correlation

Cross-trait LD score regression [17] was used to estimate the genetic correlation between ALS and MDD. SNPs were matched across the two sets of summary statistics using a flexible strategy: chromosome-and-position identifiers served as the primary key (with liftover applied when genome builds differed), and rsIDs were used as a fallback. Allele coding was harmonized to resolve strand ambiguities and allele flips. After quality control, 5,644,634 SNPs remained in the overlapping set. The genetic correlation itself was estimated as the covariance of SNP effects divided by the square root of the product of the two heritabilities, with standard errors obtained through block jackknife resampling over 200 blocks. We also monitored the intercept term throughout, since a non-zero intercept can flag residual sample overlap between the two GWAS that might bias the estimate. This analysis gave us a direct, genome-wide measure of polygenic sharing between ALS and MDD, which we could then set alongside the more granular, pathway-focused results from the other methods.

## Results

MAGMA gene-based and gene-set analyses

MAGMA [14] was applied to the ALS GWAS summary statistics from the Project MinE consortium [6], a dataset built from 27,205 cases and 110,881 controls, yielding an effective sample size of 87,381 once harmonization was complete. Across 10.4 million SNPs annotated to 18,222 protein-coding genes, using a window of 35 kb upstream and 10 kb downstream, 31 genes crossed the genome-wide significance threshold after Bonferroni correction (p < 2.74 × 10⁻⁶). The strongest hits were consistent with established ALS risk loci identified in prior GWAS [6]: C9orf72 (p = 4.42 × 10⁻¹⁴) and MOB3B (p = 3.22 × 10⁻¹⁴) on chromosome 9p21, followed by SCFD1 (p = 3.77 × 10⁻¹²) and TBK1 (p = 2.55 × 10⁻⁸). These genes encode proteins central to hexanucleotide repeat expansion biology and autophagy regulation, respectively, and their appearance at the top of the gene-based results provides internal validation of the analytic pipeline against known ALS genetics.

The gene-set analysis (Table 1) drew on eight pathways assembled from the motor neuron disease literature [3,4]. After removing genes that appeared in more than one set, giving priority to earlier sets in the hierarchy, so that, for example, C3 was kept in synaptic pruning rather than immune, 851 unique genes remained. Competitive t-tests on mean Z-statistics were corrected across the eight sets at an alpha of 0.00625.Two pathways survived that threshold (raw competitive p = 0.0031 and p = 0.0043, respectively; see Table 1 for the corresponding Bonferroni-corrected p-values). Synaptic pruning modulators emerged with a mean Z of 0.576 and a p-value of 0.0031; within that set, 32 of the 180 tested genes (17.8%) reached nominal significance on their own, with PROS1 (p = 8.51 × 10⁻⁶) and HLA-B (p = 5.59 × 10⁻⁴) contributing the strongest individual signals. Autophagy and protein quality control also cleared the threshold (mean Z = 1.391, p = 0.0043), largely on the strength of C9orf72 and TBK1, both of which were individually genome-wide significant. The two negative control sets performed as expected: housekeeping genes returned a p-value of 0.098, and monoaminergic pathways came in at 0.71, with neither anywhere close to significance. The null results for these control sets support the specificity of the enrichment signals observed in the pruning and autophagy gene sets. Immune and RNA processing pathways did not reach significance (p = 0.115 and 0.772, respectively), though it is notable that TNIP1 (p = 4.92 × 10⁻¹⁰) stood out within the immune set as a single strong hit, suggesting a possible neuroinflammatory signal that the set-level test may have lacked statistical power to detect.

**Table 1:** MAGMA gene-set enrichment results for the ALS GWAS (effective sample size = 87,381). Note: Z-statistics are from competitive gene-set tests; p-values are Bonferroni-corrected across the 8 tested gene sets. MAGMA: Multi-marker Analysis of GenoMic Annotation; ALS: Amyotrophic Lateral Sclerosis; GWAS: Genome-Wide Association Study; FDR: False discovery rate; QC: Quality control.

Gene set	Statistical test and statistic	Genes tested	Mean Z	Median Z	Nominal p < 0.05 (n)	Bonferroni-corrected p	FDR p
Synaptic pruning	Competitive t-test; mean Z statistic	180	0.576	0.554	32	0.0246	0.0246
Autophagy/protein quality control	Competitive t-test; mean Z statistic	26	1.391	1.254	7	0.0347	0.0173
Immune/neuroinflammation	Competitive t-test; mean Z statistic	13	1.015	0.389	3	0.922	0.231
RNA processing	Competitive t-test; mean Z statistic	21	0.178	-0.056	3	1	0.772
Glutamatergic	Competitive t-test; mean Z statistic	124	0.41	0.303	16	1	0.365
Neurosteroid	Competitive t-test; mean Z statistic	168	0.41	0.341	19	1	0.378
Monoaminergic (control)	Competitive t-test; mean Z statistic	96	0.293	0.302	7	1	0.809
Housekeeping (control)	Competitive t-test; mean Z statistic	158	0.474	0.319	26	0.782	0.261

GSEA and differential GSEA (DGSEA) across ALS and MDD

Moving from MAGMA to a complementary enrichment framework, we ran GSEA [15] on both the ALS and MDD GWAS [7,8], using the same eight core pathways and an expanded panel of 115 subsets for finer-grained resolution (Table 2). In ALS, 10,000 permutations confirmed what the MAGMA results had already suggested: autophagy and protein quality control showed the strongest positive enrichment of any pathway (NES = 1.968, p = 0.0001, FDR = 0.000038), with the core MND-related autophagy subset performing nearly as well (NES = 1.881, p = 0.000279). Synaptic pruning reached moderate but significant enrichment (NES = 1.401, p = 0.0068), and when broken into subsets, MHC class I molecules (NES = 1.692, p = 0.0053) and complement genes (NES = 1.469, p = 0.049) stood out. RNA processing and glutamatergic signaling, by contrast, were unremarkable in ALS (NES values below 1.07, p values above 0.36).

**Table 2:** GSEA normalized enrichment scores (NES) and p-values for major MND pathways in ALS and MDD. ALS: Amyotrophic Lateral Sclerosis; MDD: Major Depressive Disorder; GSEA: Gene Set Enrichment Analysis; NES: Normalized Enrichment Score; QC: Quality Control; MND: Motor Neuron Disease; TNF: Tumor Necrosis Factor.

Gene set	ALS NES (p)	MDD NES (p)	Leading-edge highlights (ALS)
Autophagy/protein quality control	1.968 (0.0001)*	0.809 (0.801)	C9orf72, TBK1, OPTN
Synaptic pruning	1.401 (0.0068)*	1.415 (0.0001)*	HLA-B, CX3CR1, PROS1
Immune/neuroinflammation	1.433 (0.062)	0.722 (0.862)	TNIP1, TNF
RNA processing	0.814 (0.735)	0.806 (0.786)	ASCC1, G3BP1
Glutamatergic	1.066 (0.361)	1.193 (0.054)	PRKCB, HOMER2
Neurosteroid	1.095 (0.287)	1.052 (0.318)	CYP27A1, HSD11B2
Monoaminergic (control)	0.829 (0.795)	1.082 (0.283)	VAMP2
Housekeeping (control)	1.325 (0.028)*	0.986 (0.557)	HSPA1A, PKM

The MDD picture looked quite different. Here, the strongest pathway-level signal belonged to synaptic pruning (NES = 1.415, p = 0.0001, FDR = 0.0466), carried primarily by cell-adhesion genes (NES = 1.622, p = 0.0064) and a subset of disease-associated pruning genes (NES = 1.690, p = 0.0014). Of note, enrichment of heat shock proteins, one of our housekeeping control subsets, was observed in MDD (NES = 1.702, p = 0.000831), which is consistent with the broader literature on stress-response biology in depression [21]. The most notable divergence from ALS was what happened with autophagy in MDD: the protein-aggregation/proteostasis subset actually showed depletion (NES = -1.588, p = 0.028), a clear departure from the ALS pattern and a result that highlights how differently the two disorders engage this biology.

To ask directly whether any pathway was significantly more enriched in one disorder than the other, we ran differential GSEA (DGSEA) across all pairwise combinations of the eight major sets between ALS and MDD, following the cross-disorder approach described by Gandal MJ et al. [10]. None of these 56 differential comparisons survived correction (all p_Diff > 0.05). This null result at the differential level should be interpreted cautiously, as the statistical power of cross-disorder pathway comparisons is constrained by the smaller effective sample size of the ALS GWAS relative to that of the MDD GWAS. Descriptively, the pruning enrichment scores were strikingly similar across the two disorders (NES = 1.401 in ALS versus 1.415 in MDD), consistent with the idea that microglia-mediated synaptic pruning represents a shared substrate rather than something owned by one condition or the other. Autophagy, by contrast, showed a pronounced asymmetry: the difference in NES between ALS and MDD reached 1.159 in favor of ALS, the largest observed for any pathway.

In summary, these results show a pattern in which synaptic pruning appears to be a modest but real shared substrate, emerging in both disorders at nearly identical strength. Autophagy enrichment was observed predominantly on the ALS side, while MDD showed signals in stress-response and immune-modulation pathways. The differential tests did not reach statistical significance after correction, meaning that the observed asymmetry between disorders at the pathway level should be regarded as exploratory rather than confirmatory. The qualitative split, autophagy genes among the most strongly enriched in ALS and essentially flat or depleted in MDD, is descriptively consistent with the hypothesized model but requires replication in independent or larger samples.

TWAS using S-PrediXcan

With pathway-level signals established through the MAGMA and GSEA analyses, we wanted to see how those signals translated into actual gene expression differences across tissues. S-PrediXcan [16], paired with GTEx v8 MASHR prediction models [20], allowed us to impute genetically regulated expression for thousands of genes in 14 tissues chosen for their relevance to motor and mood pathology, several brain regions, including frontal cortex, hippocampus, and substantia nigra, along with spinal cord and skeletal muscle. The ALS side used the Project MinE GWAS [6] (effective N = 87,381), and the MDD side relied on the most recent PGC meta-analysis (N = 829,249) [7,8]. For each of the eight gene sets (roughly 1,000 unique genes altogether), we tested whether target genes carried stronger expression associations than background genes, using Mann-Whitney U tests on absolute Z-scores, with false discovery rate correction applied separately within each tissue.

In ALS, the autophagy and protein quality control set dominated the results (Table 3). It showed 1.5-fold enrichment over the genomic background (Mann-Whitney p = 2.10 × 10⁻¹⁴) and produced five genes that survived FDR correction across multiple tissues. C9orf72 was the strongest single hit in the entire analysis, reaching a Z-score of 13.43 in the hypothalamus (p = 3.93 × 10⁻⁴¹). TBK1 followed in the hippocampus (Z = 7.37, p = 1.66 × 10⁻¹³). These genes have well-established roles in ALS through repeat expansion biology and autophagy regulation, respectively [3,5,6]; their emergence as the strongest TWAS signals provides convergent support for the pathway-level findings. Synaptic pruning genes showed a more modest but still consistent pattern (1.05-fold enrichment, p = 0.010), with HLA-B reaching FDR significance in the putamen (Z = -3.96, p = 7.59 × 10⁻⁵). The immune and RNA processing sets each had individual genes that reached notable significance, TNIP1 in whole blood (Z = -4.61) for immune, DDX46 in tibial nerve for RNA, but neither set cleared the correction threshold with the same confidence as autophagy. Housekeeping genes, serving as our negative control, showed little movement (1.03-fold, p = 0.334).

**Table 3:** TWAS enrichment results for major gene sets in ALS (Project MinE GWAS). Note: Enrichment is based on comparisons of absolute Z-scores; tissues are from GTEx v8; p-values are uncorrected for set-level testing. FDR: False Discovery Rate; QC: Quality Control; Z: Z score; p: Probability value; ALS: Amyotrophic Lateral Sclerosis.

Gene set	Statistical test and statistic	Genes tested	FDR-significant (n)	Enrichment ratio	Mann-Whitney p	Top gene (tissue, Z)
Autophagy/protein quality control	Mann-Whitney U test; enrichment ratio from absolute Z-scores	67	5	1.5	2.10 × 10⁻14	C9orf72 (Hypothalamus, 13.43)
Synaptic pruning	Mann-Whitney U test; enrichment ratio from absolute Z-scores	166	1	1.05	0.01	HLA-B (Putamen, -3.96)
RNA processing	Mann-Whitney U test; enrichment ratio from absolute Z-scores	61	1	1.09	0.018	DDX46 (Nerve, -3.94)
Immune/neuroinflammation	Mann-Whitney U test; enrichment ratio from absolute Z-scores	71	2	1.09	0.011	TNIP1 (Blood, -4.61)
Glutamatergic	Mann-Whitney U test; enrichment ratio from absolute Z-scores	115	2	0.96	0.895	PRKCB (Nerve, -4.70)
Neurosteroid	Mann-Whitney U test; enrichment ratio from absolute Z-scores	142	2	0.94	1	CYP27A1 (Cerebellum, -4.94)
Monoaminergic (control)	Mann-Whitney U test; enrichment ratio from absolute Z-scores	83	0	0.85	1	VAMP2 (Cingulate, -3.41)
Housekeeping (control)	Mann-Whitney U test; enrichment ratio from absolute Z-scores	151	6	1.03	0.334	NDUFA2 (Nerve, -4.59)

The MDD results were broader and more diffuse, consistent with a condition with higher polygenicity and a much larger discovery sample (Table 4). The glutamatergic set was the only one that edged into significance at the set level (1.04-fold enrichment, p = 0.025), driven by EP300 in the cortex (Z = 6.98, p = 2.94 × 10⁻¹²) and PRKAR2A in tibial nerve (Z = 5.86). Pruning genes actually produced more individual FDR-significant hits in MDD than in ALS, 19 across the set, including RHOA in skeletal muscle (Z = 9.07) and SDK1 in blood (Z = -5.83), but the overall set-level enrichment was weaker (1.02-fold, p = 0.696), suggesting a real but scattered signal rather than the concentrated effect observed in ALS autophagy. Neurosteroid and monoaminergic pathways each had their standout genes, such as CYP2D6 in the cerebellum and DRD2 in the cerebellar hemisphere, but without strong set-level support. On the MDD side, autophagy genes produced only three FDR-significant hits and showed slight depletion rather than enrichment (0.91-fold, p = 0.998). This gap between the two disorders, dominant autophagy in ALS, essentially flat in MDD, was consistent across all analytic methods.

**Table 4:** TWAS enrichment results for major gene sets in MDD (PGC 2025 GWAS). Note: The same conventions as in Table 3 were used; the larger sample size in MDD yielded more individual hits overall. FDR: False Discovery Rate; Z: Z score; p: probability value; QC: Quality Control; MDD: Major Depressive Disorder; GWAS: Genome-Wide Association Study; PGC: Psychiatric Genomics Consortium.

Gene set	Statistical test and statistic	Genes tested	FDR-significant (n)	Enrichment ratio	Mann-Whitney p	Top gene (tissue, Z)
Glutamatergic	Mann-Whitney U test; enrichment ratio from absolute Z-scores	112	8	1.04	0.025	EP300 (Cortex, 6.98)
Synaptic pruning	Mann-Whitney U test; enrichment ratio from absolute Z-scores	166	19	1.02	0.696	RHOA (Muscle, 9.07)
Monoaminergic	Mann-Whitney U test; enrichment ratio from absolute Z-scores	78	9	1.09	0.234	DRD2 (Cerebellar hemisphere, -6.22)
Neurosteroid	Mann-Whitney U test; enrichment ratio from absolute Z-scores	144	19	1.03	0.407	CYP2D6 (Cerebellum, -7.00)
Immune/neuroinflammation	Mann-Whitney U test; enrichment ratio from absolute Z-scores	71	6	0.99	0.378	TLR9 (Muscle, 4.73)
RNA processing	Mann-Whitney U test; enrichment ratio from absolute Z-scores	63	3	0.92	0.971	METTL14 (Cortex, -6.78)
Housekeeping (control)	Mann-Whitney U test; enrichment ratio from absolute Z-scores	152	20	0.97	0.98	MDH2 (Blood, 5.72)
Autophagy/protein quality control	Mann-Whitney U test; enrichment ratio from absolute Z-scores	67	3	0.91	0.998	OPA1 (Putamen, -4.13)

When we looked across the two disorders for shared threads, synaptic pruning stood out as the most consistent bridge. MHC-related genes like HLA-B and HLA-C appeared in both sets of results, as did microglial regulators, though the MDD signal showed more polygenic scatter, a natural consequence of the larger sample picking up smaller effects spread across more genes. The absence of autophagy enrichment in MDD reinforced the pattern observed at every level of analysis: the two disorders appear to share a pruning-related signal, but each is associated with a different downstream pathway profile. In ALS, that profile centers on protein aggregation and autophagy collapse; in MDD, it looks more like stress-responsive and immune-related expression changes [10]. The TWAS results added a tissue-specific dimension, autophagy genes showed the strongest signals in motor-relevant regions such as the spinal cord and hippocampus in ALS, while MDD’s glutamatergic signal was concentrated in cortical and limbic tissues, the regions most consistently implicated in mood regulation.

In sum, the TWAS results added a layer of tissue-level specificity to the broader pattern. Shared microglial and pruning-related expression changes appeared in both disorders, but the way each condition engaged those signals was different, autophagy collapse was concentrated in motor-relevant tissues in ALS, whereas glutamatergic signals were centered in limbic circuits in MDD.

LD score regression: partitioned heritability and genetic correlations

The final analytic layer examined the distribution of common-variant heritability across the genome. Partitioned LD score regression [18] allowed us to ask, for each of the eight gene sets, whether the SNPs falling within those annotations carried more of the heritable risk than their genomic footprint would predict. We used European-ancestry LD scores from the 1000 Genomes Project [19] and tested one-tailed for positive enrichment, which is the standard approach when the hypothesis is directional [18].

In ALS [6], autophagy and protein quality control genes stood well above everything else (Table 5). That set showed 2.20-fold enrichment in LD-adjusted heritability (one-tailed p = 1.01 × 10⁻⁵), which was the single strongest partitioned signal observed for either disorder. The annotated SNPs had a mean chi-squared value of 2.49, close to double the background level, and the enrichment persisted after LD correction. RNA processing came in with 1.28-fold enrichment (p = 0.0016) and immune genes at 1.77-fold (p = 0.0096), both statistically significant signals, though neither on the same scale as autophagy. Synaptic pruning modulators showed modest enrichment of 1.03-fold (p = 0.187) that did not reach significance at the set level; however, the Mann-Whitney test on per-SNP contributions was very small (p = 5.68 × 10⁻¹⁷), pointing to a broad, low-level polygenic push spread across many variants rather than a few large effects concentrated in one or two genes. Glutamatergic and neurosteroid pathways were essentially flat, consistent with their weak showing in the upstream analyses. The two control sets, housekeeping and monoaminergic, stayed near the null (both p > 0.14), consistent with the expected behavior of negative controls.

**Table 5:** LDSC partitioned heritability results for ALS (one-tailed enrichment tests). Note: Enrichment was estimated using partitioned LDSC; p-values are one-tailed for the positive enrichment hypothesis. LDSC: Linkage Disequilibrium Score Regression; ALS: Amyotrophic Lateral Sclerosis; SNPs: Single-Nucleotide Polymorphisms; LD: Linkage Disequilibrium; Adj.:  Adjusted; p: Probability value; QC: Quality Control; n: Number.

Gene set	Statistical test and statistic	Genes (n)	Annotated SNPs (n)	% of SNPs	LD-adjusted enrichment	One-tailed p	Mann-Whitney p
Autophagy/protein quality control	Partitioned LD score regression; LD-adjusted enrichment	37	7,819	0.08	2.2	1.01 × 10⁻⁵	1.56 × 10⁻⁸³
RNA processing	Partitioned LD score regression; LD-adjusted enrichment	33	4,441	0.05	1.28	1.56 × 10⁻³	5.06 × 10⁻¹¹
Immune/neuroinflammation	Partitioned LD score regression; LD-adjusted enrichment	18	6,153	0.07	1.77	9.59 × 10⁻³	2.51 × 10⁻²
Synaptic pruning	Partitioned LD score regression; LD-adjusted enrichment	192	111,952	1.19	1.03	0.187	5.68 × 10⁻¹⁷
Glutamatergic	Partitioned LD score regression; LD-adjusted enrichment	127	70,309	0.75	1.2	0.558	6.38 × 10⁻⁴
Monoaminergic (control)	Partitioned LD score regression; LD-adjusted enrichment	101	30,743	0.33	1.32	0.141	1.02 × 10⁻⁴
Housekeeping (control)	Partitioned LD score regression; LD-adjusted enrichment	182	21,304	0.23	1.21	0.506	5.90 × 10⁻²⁷
Neurosteroid	Partitioned LD score regression; LD-adjusted enrichment	183	3,930	0.04	0.95	0.994	1

MDD showed a different heritability profile, though it still pointed back to pruning as a connecting thread (Table 6). RNA processing led the way here, with 1.48-fold enrichment (p = 7.44 × 10⁻⁷), followed by synaptic pruning at 1.32-fold (p = 0.00014) and immune genes at 1.89-fold (p = 0.002). This trio, RNA, pruning, and immune, is consistent with the MDD genetic architecture as it has been described in recent large-scale work: a condition shaped by regulatory and stress-responsive biology rather than protein-clearance mechanisms [7,8]. Autophagy genes were actually depleted in MDD heritability (0.74-fold, p = 1.00), a finding that was consistent with the results from every other method applied in this study. The control sets in MDD also behaved well, staying near the null.

**Table 6:** LDSC partitioned heritability results for MDD (one-tailed enrichment tests). LDSC: Linkage Disequilibrium Score Regression; MDD: Major Depressive Disorder; LD: Linkage Disequilibrium; Adj.: Adjusted; SNPs: Single-Nucleotide Polymorphisms; p: Probability value; QC: Quality Control; n: Number.

Gene set	Statistical test and statistic	Genes (n)	Annotated SNPs (n)	% of SNPs	LD-adjusted enrichment	One-tailed p	Mann-Whitney p
RNA processing	Partitioned LD score regression; LD-adjusted enrichment	33	3,315	0.05	1.48	7.44 × 10⁻⁷	2.02 × 10⁻⁵⁵
Synaptic pruning	Partitioned LD score regression; LD-adjusted enrichment	192	79,533	1.14	1.32	1.40 × 10⁻⁴	4.44 × 10⁻⁹⁰
Immune/neuroinflammation	Partitioned LD score regression; LD-adjusted enrichment	18	4,334	0.06	1.89	2.01 × 10⁻³	3.25 × 10⁻¹⁰
Monoaminergic	Partitioned LD score regression; LD-adjusted enrichment	101	23,587	0.34	1.37	0.23	4.15 × 10⁻¹
Glutamatergic	Partitioned LD score regression; LD-adjusted enrichment	127	51,316	0.74	1.18	0.708	9.60 × 10⁻¹
Neurosteroid	Partitioned LD score regression; LD-adjusted enrichment	183	2,857	0.04	0.98	0.784	9.98 × 10⁻¹
Housekeeping (control)	Partitioned LD score regression; LD-adjusted enrichment	182	16,611	0.24	1.03	0.839	1
Autophagy/protein quality control	Partitioned LD score regression; LD-adjusted enrichment	37	5,827	0.08	0.74	1	1

The final piece was the cross-trait genetic correlation, estimated through bivariate LDSC on the ALS and MDD summary statistics. The result was small and negative (rg = -0.044, SE = 0.034, p = 0.196), for practical purposes, indistinguishable from zero. This is broadly in line with what others have reported for ALS and psychiatric phenotypes [9]. The intercept was close to 1.0, so there was no sign of meaningful sample overlap between the two GWAS distorting the estimate. A near-zero genome-wide correlation indicates that, in aggregate, the common-variant architectures of ALS and MDD do not covary. This does not preclude the possibility of convergence at the level of individual biological pathways, which is the pattern suggested by the pathway-level analyses reported above. The implications of this dissociation between genome-wide and pathway-level findings are addressed in the Discussion.

## Discussion

Autophagy and the cellular cleanup crisis in ALS

When the results from all four analytic methods were considered together, the most prominent pattern was the degree of consistency across independent approaches, and how closely they tracked what has been observed about these diseases in prior work. The TWAS findings crystallized a central theme: ALS is not just about motor neurons dying. It is about motor neurons whose internal waste-disposal machinery has given out. The emergence of C9orf72 in the hypothalamus with a Z-score of 13.43 (p = 3.93 × 10⁻⁴¹) was consistent with its established role as the most common genetic cause of ALS, but seeing TBK1 follow closely behind in the hippocampus (Z = 7.37) reinforced the autophagy narrative as more than one interpretation among several. These are the genomic echoes of protein aggregates piling up because the autophagosome-lysosome system cannot keep pace, and they fit closely with what Antonakoudis A et al. [3] outlined in their recent review of motor neuron disease mechanisms, in which autophagy defects occupy a central role in both sporadic and familial forms and are often worsened by environmental stressors that a healthy neuron would normally be able to absorb.

The polygenic architecture beneath the surface

The partitioned heritability results from LDSC took that story and made it polygenic in a way that felt important. The 2.20-fold enrichment in the autophagy gene set for ALS (one-tailed p = 1.01 × 10⁻⁵) was not being carried by one or two famous loci dragging up the average. The annotated SNPs had a mean chi-squared of 2.49, nearly double the background, which means that across many common variants, each individually small, there is a steady accumulation of risk pointing toward protein mishandling in motor neurons. MDD’s heritability profile was a different animal entirely. RNA processing led at 1.48-fold enrichment (p = 7.44 × 10⁻⁷), immune and neuroinflammatory genes came in at 1.89-fold (p = 0.002), and autophagy was not just absent but actively depleted (0.74-fold, p = 1.00). That asymmetry captured something that rings true both clinically and biologically: ALS as a protein-clearance catastrophe, MDD as a chronic stress response that has become stuck in a self-reinforcing loop. And threading through both conditions, the synaptic pruning set showed up as the quiet connector, 1.03-fold in ALS, 1.32-fold in MDD, consistent with the idea that microglial over-pruning might serve as a shared vulnerability, whether driven by complement activation in motor neuron disease or by aberrant synaptic remodeling in depression [4,11]. The housekeeping control genes, meanwhile, stayed flat in both disorders across every method, which is exactly what needed to happen for us to trust that the other signals were real.

The pruning continuum: a model that emerged from the data

**Figure 1:**
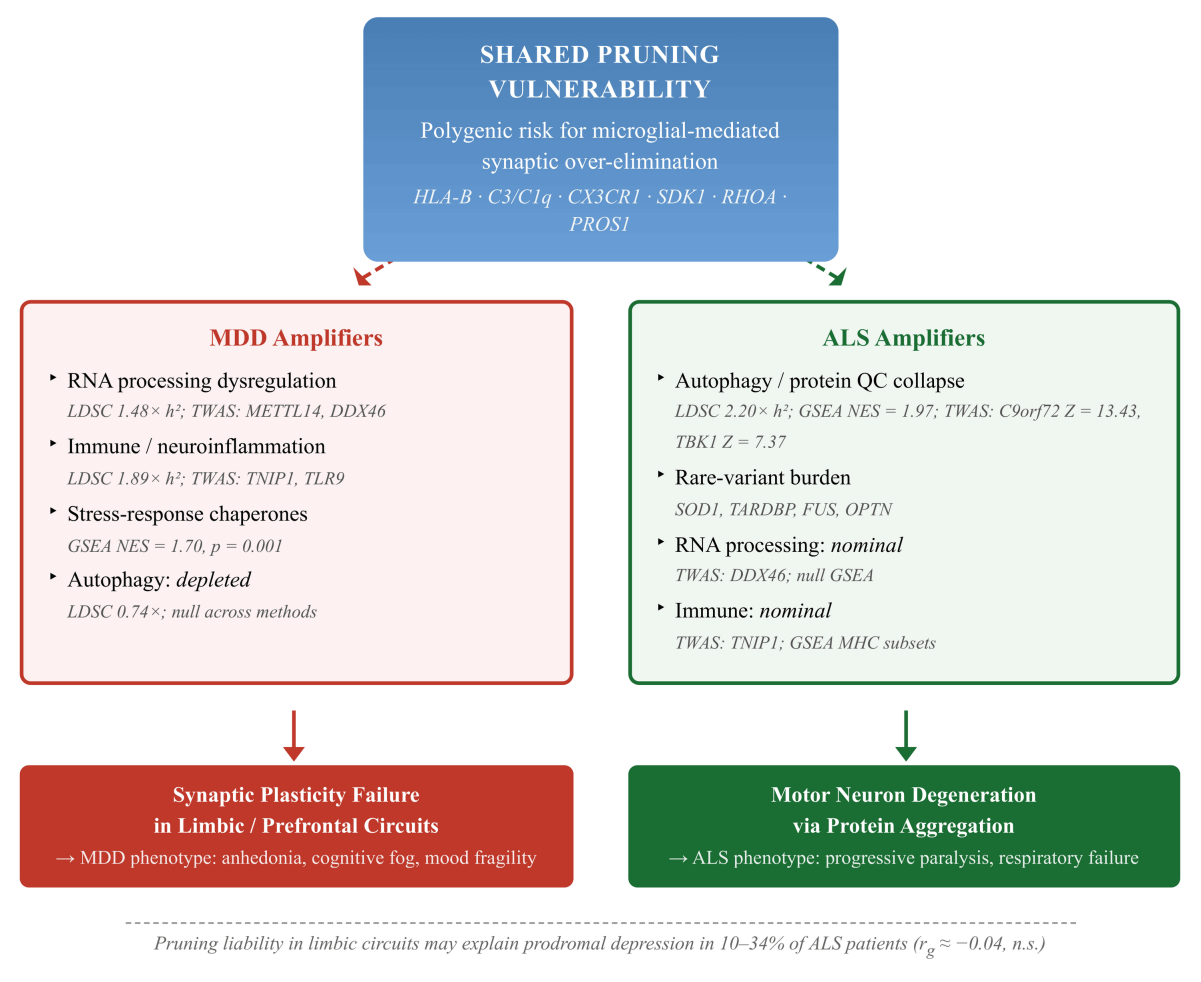
The microglial pruning continuum model.

The hypothesis that kept tying everything together was this notion of a microglial pruning continuum, a shared biological entry point at which microglia become overenthusiastic about stripping synapses, creating vulnerability for both MDD and ALS, with the two conditions then peeling apart depending on what goes wrong downstream (Figure 1). We did not arrive at this idea before looking at the data and then go hunting for confirmation. It came together gradually as the same MHC and complement genes kept turning up across analyses, HLA-B in the putamen for ALS, SDK1 and RHOA in MDD, pointing to microglia as the common actor rather than passive bystanders [6,7]. That convergence makes intuitive sense when viewed against the comorbidity numbers: depression rates in ALS range between 10% and 34%, often appearing early enough in the disease course that a purely reactive explanation feels incomplete [1,2]. Under this framework, those early mood changes become interpretable as prodromal pruning liability spilling into limbic and prefrontal circuits before the motor system takes the brunt of the damage, which fits with what clinicians have observed in patients who describe a vague fog or loss of motivation months before any weakness shows up [22]. The point is not that pruning causes both diseases in equal measure. It is that pruning loads the gun, and what pulls the trigger is different on each side.

The asymmetry is where the model earns its keep. In ALS, autophagy did not just show enrichment, it overwhelmed every other pathway, with C9orf72 and TBK1 standing out in the TWAS and GSEA results and a 2.2-fold LDSC signal that left little room for doubt about protein clearance failure being central [5,6]. In MDD, autophagy was depleted or flat wherever we looked, and instead RNA processing and immune regulation stepped forward, METTL14 and DDX46 in cortical tissues, TNIP1 and TLR9 elsewhere, sketching a picture of a system that is constantly adjusting itself under stress rather than breaking down outright [13,21]. That split maps onto what we see every day in the clinic: ALS as a relentless piling up of toxic waste, MDD as a stress response that never quite returns to baseline. The continuum model holds these together without pretending the overlap is genome-wide, and the near-zero genetic correlation (rg around -0.04, p = 0.196) actually fits neatly if the shared pruning thread is modest in size and gets drowned out by strong disorder-specific amplifiers pulling in opposite directions [9,10].

However, this model must be interpreted within the constraints of the evidence. The genome-wide genetic correlation between ALS and MDD is near zero, which means that, at the level of aggregate polygenic architecture, these conditions do not share substantial common-variant liability. The pathway-level convergence we observed, particularly in synaptic pruning, exists within that context of overall genetic independence and may reflect a modest biological overlap rather than a fundamentally shared etiology. Furthermore, the differential GSEA comparisons did not survive correction for multiple testing, meaning that the apparent disorder-specific enrichment patterns (autophagy in ALS, RNA/immune in MDD) are descriptive and exploratory rather than statistically confirmed divergences. Future studies with larger ALS GWAS sample sizes will be needed to determine whether these asymmetries reach formal significance.

If this framework proves durable, it changes how we think about both conditions, not as isolated diagnostic categories, but as points on a biological spectrum where circuit vulnerability and the balance of genetic load together determine what clinical picture eventually emerges. For someone carrying high pruning polygenic risk who goes on to develop ALS, the early mood symptoms might represent the first expression of that shared liability rather than an adjustment reaction to the diagnosis. It also opens practical doors: complement modulators aimed at dialing back pruning could potentially help mood symptoms in ALS subgroups, while autophagy-boosting compounds would need to be handled carefully in MDD to avoid disrupting synaptic remodeling that is already fragile [11,12]. The model is not without gaps, pruning signals were stronger in MDD and more nominal in ALS, and we cannot yet separate causal contributions from correlational ones, but it provides a scaffold that makes the comorbidity data make sense in a way that earlier frameworks have not managed to do [23,24].

As illustrated in Figure 1, the proposed microglial pruning continuum model suggests that genetic variants regulating synaptic pruning (e.g., HLA and complement pathways) create a shared vulnerability mediated by microglia, which then diverges into distinct clinical phenotypes depending on disorder-specific biological amplifiers: autophagy failure driving motor neurodegeneration in ALS, and RNA and immune dysregulation driving synaptic plasticity deficits in MDD. Within this framework, the high prevalence of early depressive symptoms in ALS may reflect pruning-related effects in limbic circuits before overt motor neuron loss.

Clinical implications

The finding that kept circling back was how naturally these results could change conversations that already happen in clinic every week, especially with the ALS patient who brings up the low mood or mental fog that started well before the first twitch. We have always leaned heavily on the adjustment explanation, and sometimes that is all it is. But seeing the pruning signals line up across both disorders makes you wonder whether, in at least a subset of cases, that early depression is the first biological whisper of the same microglial overactivity that will eventually chew through motor synapses [1]. For the 10% to 34% of ALS patients who develop depression, this reframes the comorbidity as something potentially worth screening for with pruning-related polygenic risk scores, not only as a quality-of-life concern, but as a possible window into how aggressively the motor disease itself might progress, particularly when autophagy modifiers are also part of that person’s genetic picture [2,25].

On the ALS side specifically, the dominance of autophagy handed us something that feels closer to a concrete therapeutic direction than most genomic studies manage to point toward. The C9orf72 and TBK1 signals were not gesturing vaguely at biology; they were pointing to why protein aggregates accumulate so relentlessly in these patients, and why drugs that nudge autophagy, rapamycin analogs, TBK1 activators, might actually buy meaningful time if they can be deployed before motor neurons start dropping off in earnest [5,6]. But the pruning overlap complicates that picture in a way that is ultimately useful rather than frustrating. If we begin modulating microglia to slow neurodegeneration, we might inadvertently help or worsen the mood symptoms that so many of these patients struggle with. That pushes us toward genuinely multidisciplinary care, neurologists, psychiatrists, and immunologists thinking about the same patient together, particularly when someone’s autophagy pathway is failing but their pruning liability is also high. The near-zero genome-wide genetic correlation (rg = -0.044) is actually reassuring on one front: it means we are not dealing with blanket shared risk that would muddy individual prediction. But the pathway convergence suggests that targeted trials could still yield cross-disorder benefits, such as complement inhibitors for the subset of ALS patients presenting with prominent early mood changes [11,12].

For MDD, the RNA processing and immune enrichments point toward something we may have been undershooting with standard treatment in many cases. The METTL14 and TNIP1 hits suggest a system that is chronically misfiring under stress, epitranscriptomic adjustments and low-grade inflammation that SSRIs were never designed to reach [13,21]. The pruning signal in MDD feels actionable in its own right: microglial modulators currently being trialed in neurodegenerative contexts could have crossover potential, perhaps rescuing synaptic density in the prefrontal circuits that keep getting stripped back in recurrent depression. It also makes you rethink why certain patients respond to plasticity-promoting agents like ketamine, or to combination approaches that layer in glutamatergic and anti-inflammatory effects [26,27]. If pruning really is a shared thread, those drugs might not just be lifting mood, they could be stabilizing the very synapses that microglia are over-clearing in both conditions. In ALS specifically, that raises the tantalizing possibility that preserving motor synapses might be within reach if the autophagy system can be kept from collapsing at the same time [5].

The broader takeaway for clinical practice is that comorbidity in motor neuron disease is not random scatter. The 65% multimorbidity rate that Glasmacher SA et al. [25] reported reflects the pruning and immune hits bleeding into cardiovascular strain, respiratory vulnerability, and the persistent low mood that shadows so many patients through the course of their illness [28]. For families, this means we can offer something more honest than vague reassurance: here is a shared biological thread, here is where the two conditions diverge, and here is what is being targeted. It does not solve everything, but it provides a map for designing trials that go after the underlying biology rather than just managing symptoms, and possibly a way to intervene earlier in the patients who sit at that difficult intersection.

However, we emphasize that the clinical implications discussed here, including PRS stratification, complement modulator therapy, and microglial-targeted interventions, remain speculative and are not directly supported by the observational genomic design of this study. No experimental validation or clinical outcome data were generated. These suggestions should be understood as hypotheses for future investigation rather than actionable clinical recommendations.

Limitations

Several limitations of this study should be acknowledged explicitly. First, the eight biological gene sets were manually curated by the investigator and, while informed by published databases and disease-specific reviews [3,4,5,6,7,8], were not derived from a single standardized source. The hierarchical procedure used to resolve gene overlap across sets, always prioritizing the set higher in the predetermined order, may have influenced enrichment results by removing potentially relevant genes from lower-priority sets. Although this approach was applied consistently and transparently, it introduces a degree of subjectivity that could affect replicability. Full gene lists and the curation rationale are provided in Appendix 1 to facilitate independent evaluation.

Second, the differential GSEA comparisons between ALS and MDD did not survive correction for multiple testing across 56 pairwise tests. This means that the apparent disorder-specific enrichment patterns should be treated as exploratory observations rather than confirmed divergences. The limited effective sample size of the ALS GWAS (N = 87,381), relative to MDD (N = 829,249), likely contributed to reduced power for cross-disorder comparisons.

Third, the study relies entirely on previously published GWAS summary statistics and computational imputation of gene expression (S-PrediXcan). No individual-level genotype data, functional assays, or clinical outcome data were used. As a result, all findings are associational and cannot establish causal or mechanistic relationships between pathways and disease phenotypes.

Fourth, the simplified fallback procedure used for LDSC partitioned heritability in cases of numerical instability deviates from the standard implementation and, while conservative in design, has not been independently validated. Results from affected annotations should therefore be interpreted with corresponding caution.

Fifth, the near-zero genome-wide genetic correlation between ALS and MDD (rg = -0.044, p = 0.196) places a ceiling on how strongly shared biology can be inferred. The pathway-level convergence we observed, particularly for synaptic pruning, exists within a context of overall genetic independence between the two disorders. This dissociation may reflect genuine biological complexity (i.e., small shared pathways embedded within largely non-overlapping genetic architectures), but it also means that the continuum model should be regarded as a hypothesis-generating framework rather than a confirmed biological relationship.

Finally, no replication dataset was used to independently validate the pathway enrichment findings. While the convergence across four independent analytic methods (MAGMA, GSEA, TWAS, LDSC) provides internal cross-validation, external replication in independent ALS and MDD cohorts will be essential to confirm the robustness and generalizability of these results.

Robustness, novelty, and what comes next

What gave us confidence in the overall picture was not any single dramatic finding, but the way signals kept converging across methods that approach the question from fundamentally different angles. The MAGMA gene-set hits, the TWAS tissue-specific patterns, and the LDSC heritability enrichments, none of these were selected to agree with one another, and yet they converged on the same story: pruning as the quiet thread running through both disorders, with autophagy entering the picture only on the ALS side. We built the housekeeping and monoaminergic control sets into the analysis specifically to catch ourselves if the results were merely noise dressed up as biology, and they stayed flat throughout. That kind of internal consistency across independent analytic layers is not guaranteed in multi-method genomic projects, and its presence here matters. Even the near-zero genetic correlation did not work against the framework; it simply confirmed what the pathway results had already been suggesting, that shared biology and genome-wide overlap are not the same thing, and meaningful convergence can exist at the pathway level even when the aggregate picture looks like two unrelated conditions [9,10].

The novelty of this work was not in identifying pruning or autophagy as relevant to ALS or MDD individually. Both have been discussed in their respective literatures for years. What felt genuinely new was bringing them together in a single analytic framework and showing that they behave as asymmetric amplifiers sitting on a shared pruning foundation, and that data from multiple independent methods supported that architecture without requiring us to nudge the results in any particular direction. It reframes the comorbidity picture in a way that has not been attempted at this level of genomic detail before. The depression that affects 10% to 34% of ALS patients may not be entirely a psychological response to the diagnosis; some portion of it could be an early manifestation of the same microglial over-pruning that will eventually damage motor circuits [1,2]. And the clear separation between ALS and MDD on autophagy, dominant in one, depleted in the other, helps explain why the two disorders look so different in the clinic despite sharing a pruning substrate, without requiring anyone to force a “one disease” story that the data clearly do not support [5,7].

## Conclusions

Where this goes next matters as much as what we found. The model is testable, and it needs to be tested prospectively. Longitudinal cohorts that track both motor and mood trajectories alongside genomic and transcriptomic profiling could tell us whether pruning polygenic risk actually predicts early depression in ALS, or whether the association we see here is correlational rather than causal. We also need better-powered differential GSEA, our pairwise comparisons did not survive correction, likely because of sample size limitations rather than a genuine absence of pathway-level differences. And the therapeutic implications, while exciting, remain speculative until someone runs the trials: complement inhibitors in ALS patients with prominent mood symptoms, microglial modulators in treatment-resistant MDD, autophagy enhancers with careful monitoring of synaptic remodeling. None of that is ready for the prescription pad tomorrow, but the biology now points somewhere specific rather than everywhere at once.

This work does not rewrite the textbooks overnight, and we would not claim that it does. But it moves us a step closer to seeing ALS and MDD as points on a shared biological spectrum rather than as conditions that happen to co-occur by chance. Microglial pruning sets the stage, and disorder-specific amplifiers determine the clinical outcome, the data held together, the model feels ready for prospective testing, and if even a fraction of it translates into better care for the people who live at the intersection of motor loss and mood fragility, it will have been worth the effort.

## Appendices

Appendix 1

**Table 7:** Gene list by category for analysis. Note. n: number of genes per subcategory.

No.	Category / Subcategory	Gene symbols	n
1	Negative controls: housekeeping genes		
	Ribosomal large subunit	RPL3, RPL4, RPL5, RPL6, RPL7, RPL7A, RPL8, RPL9, RPL10, RPL10A, RPL11, RPL12, RPL13, RPL13A, RPL14, RPL15, RPL17, RPL18, RPL18A, RPL19, RPL21, RPL22, RPL23, RPL23A	24
	Ribosomal small subunit	RPS2, RPS3, RPS3A, RPS4X, RPS4Y1, RPS5, RPS6, RPS7, RPS8, RPS9, RPS10, RPS11, RPS12, RPS13, RPS14, RPS15, RPS15A, RPS16, RPS17, RPS18, RPS19, RPS20, RPS21	23
	Cytoskeletal actin	ACTB, ACTG1, ACTA1, ACTA2, ACTC1, ACTN1, ACTN2, ACTN4, PFN1, PFN2	10
	Cytoskeletal tubulin	TUBA1A, TUBA1B, TUBA1C, TUBA3C, TUBA3D, TUBA4A, TUBA8, TUBB, TUBB1, TUBB2A, TUBB2B, TUBB3, TUBB4A, TUBB4B, TUBB6	15
	Glycolysis	HK1, HK2, GPI, PFKM, PFKP, PFKL, ALDOA, ALDOB, ALDOC, TPI1, GAPDH, PGK1, PGK2, PGAM1, PGAM2, ENO1, ENO2, ENO3, PKM, PKLR, LDHA, LDHB, LDHC	23
	TCA cycle	CS, ACO1, ACO2, IDH1, IDH2, IDH3A, IDH3B, IDH3G, OGDH, SUCLA2, SUCLG1, SUCLG2, SDHA, SDHB, SDHC, SDHD, FH, MDH1, MDH2	19
	Mitochondrial complex I	NDUFA1, NDUFA2, NDUFA3, NDUFA4, NDUFA5, NDUFA6, NDUFA7, NDUFA8, NDUFA9, NDUFA10, NDUFA11, NDUFA12, NDUFA13, NDUFB1, NDUFB2, NDUFB3, NDUFB4, NDUFB5, NDUFB6	19
	Mitochondrial complex IV	COX4I1, COX4I2, COX5A, COX5B, COX6A1, COX6A2, COX6B1, COX6B2, COX6C, COX7A1, COX7A2, COX7A2L, COX7B, COX7C, COX8A, COX8C	16
	ATP synthase	ATP5F1A, ATP5F1B, ATP5F1C, ATP5F1D, ATP5F1E, ATP5MC1, ATP5MC2, ATP5MC3, ATP5MD, ATP5ME, ATP5MF, ATP5MG, ATP5PB, ATP5PD, ATP5PF, ATP5PO	16
	Heat shock proteins	HSPA1A, HSPA1B, HSPA1L, HSPA2, HSPA4, HSPA4L, HSPA5, HSPA6, HSPA8, HSPA9, HSPA12A, HSPA12B, HSPA13, HSP90AA1, HSP90AB1, HSP90B1, HSPH1	17
2	Monoaminergic systems		
	Dopamine receptors	DRD1, DRD2, DRD3, DRD4, DRD5	5
	Dopamine synthesis and metabolism	TH, DDC, COMT, MAOA, MAOB, DBH, GCH1, PTS, QDPR, SPR	10
	Dopamine transport and signaling	SLC6A3, SLC18A1, SLC18A2, PPP1R1B, GNAL, ADCY5, PDE1B, PDE10A	8
	Serotonin receptors	HTR1A, HTR1B, HTR1D, HTR1E, HTR1F, HTR2A, HTR2B, HTR2C, HTR3A, HTR3B, HTR3C, HTR3D, HTR3E, HTR4, HTR5A, HTR5BP, HTR6, HTR7	18
	Serotonin synthesis and metabolism	TPH1, TPH2, SLC6A4, AANAT, ASMT	5
	Norepinephrine receptors	ADRA1A, ADRA1B, ADRA1D, ADRA2A, ADRA2B, ADRA2C, ADRB1, ADRB2, ADRB3	9
	Norepinephrine synthesis and transport	PNMT, SLC6A2	2
	Histamine system	HRH1, HRH2, HRH3, HRH4, HDC, HNMT, SLC22A3	7
	Trace amine receptors	TAAR1, TAAR2, TAAR5, TAAR6, TAAR8, TAAR9	6
	Downstream signaling	GNB1, GNB2, GNB3, GNB4, GNB5, GNG2, GNG3, GNG4, GNG7, GNG11, GNG12, ADCY1, ADCY2, ADCY7, ADCY8, ADCY9, PDE4A, PDE4B, PDE4C, PDE4D	20
	Vesicular machinery	SYT1, SYT2, SYT4, SYT7, SYT11, SNAP25, VAMP2, STX1A, STX1B, CPLX1, CPLX2	11
3	Neurosteroid pathways		
	Cholesterol supply and transport	STAR, TSPO, TSPO2, VDAC1, VDAC2, VDAC3, ACBD1, ACBD3, NPC1, NPC2, STARD1, STARD3, STARD4, STARD5, STARD6	15
	Electron transfer partners	FDX1, FDX2, FDXR, POR, CYB5A, CYB5R3	6
	Cholesterol biosynthesis (terminal)	HMGCR, HMGCS1, MVK, PMVK, MVD, IDI1, IDI2, FDPS, FDFT1, SQLE, LSS, CYP51A1, TM7SF2, MSMO1, NSDHL, HSD17B7, SC5D, DHCR7, DHCR24, EBP	20
	Side-chain cleavage	CYP11A1	1
	3β-HSD isomerase	HSD3B1, HSD3B2, HSD3B7	3
	17α-hydroxylase/lyase	CYP17A1	1
	17β-HSD family	HSD17B1, HSD17B2, HSD17B3, HSD17B4, HSD17B6, HSD17B8, HSD17B10, HSD17B11, HSD17B12, HSD17B13, HSD17B14	11
	5α-reductases	SRD5A1, SRD5A2, SRD5A3	3
	5β-reductase	AKR1D1	1
	3α-HSD (AKR1C)	AKR1C1, AKR1C2, AKR1C3, AKR1C4	4
	20α-HSD	CBR1	1
	11β-HSD	HSD11B1, HSD11B2, HSD11B1L	3
	Aromatase	CYP19A1	1
	Adrenal CYPs	CYP11B1, CYP11B2, CYP21A2	3
	Sulfotransferases	SULT1E1, SULT2A1, SULT2B1, SULT1A1	4
	Sulfatase	STS	1
	Glucuronosyltransferases	UGT1A1, UGT1A4, UGT2B7, UGT2B15, UGT2B17, UGT2B4	6
	Steroid-metabolizing CYPs	CYP7A1, CYP7B1, CYP27A1, CYP46A1, CYP39A1, CYP3A4, CYP3A5, CYP3A7, CYP2D6, CYP1A1, CYP1A2, CYP1B1	12
	Additional reductases	DHRS9, DHRS4, RDH5, WWOX, PECR	5
	Classical steroid receptors	ESR1, ESR2, AR, PGR, NR3C1, NR3C2	6
	Coregulators	NCOA1, NCOA2, NCOA3, NCOR1, NCOR2, NRIP1, PNRC1, PNRC2, PELP1	9
	Xenobiotic receptors	NR1I2, NR1I3	2
	Oxysterol receptors	NR1H3, NR1H2	2
	Transcription factors	NR5A1, NR5A2, NR0B1, GATA4, GATA6, SP1, SREBF1, SREBF2, NR4A1, NR4A2	10
	Membrane progesterone receptors	PGRMC1, PGRMC2, PAQR5, PAQR6, PAQR7, PAQR8, PAQR9	7
	Membrane estrogen receptor	GPER1	1
	Androgen-related	GPRC6A, OXER1	2
	Sigma receptors	SIGMAR1, TMEM97	2
	GABA-A receptor subunits	GABRA2, GABRA3, GABRA4, GABRA5, GABRA6, GABRB1, GABRB3, GABRD, GABRE, GABRG1, GABRG2, GABRG3, GABRP, GABRQ, GABRR1, GABRR2, GABRR3	17
	Carrier proteins	SHBG, SERPINA6, ALB, AFP, TTR	5
	Chaperones	FKBP4, FKBP5, PPID, PTGES3	4
	Transporters	ABCB1, ABCC1, ABCC4, ABCG2, SLC22A8, SLC22A6, SLCO1A2, SLCO2B1, SLC10A1, SLC10A6	10
	Endozepines	DBI	1
	Signaling kinases	SRC, MAPK1, MAPK3, PIK3R1	4
4	Glutamatergic systems		
	NMDA receptors	GRIN1, GRIN2A, GRIN2B, GRIN2C, GRIN2D, GRIN3A, GRIN3B	7
	AMPA receptors	GRIA1, GRIA2, GRIA3, GRIA4	4
	Kainate receptors	GRIK1, GRIK2, GRIK3, GRIK4, GRIK5	5
	Delta receptors	GRID1, GRID2	2
	Receptor-associated proteins	GRIP1, GRIP2, PICK1, SHANK1, SHANK2, SHANK3, HOMER1, HOMER2, HOMER3, DLGAP1, DLGAP2, DLGAP3, DLGAP4, DLG1, DLG2, DLG3, DLG4	17
	Glutamate transporters	SLC1A1, SLC1A2, SLC1A3, SLC1A6, SLC1A7, SLC17A6, SLC17A7, SLC17A8	8
	Glutamate metabolism	GLUL, GLS, GLS2, GLUD1, GLUD2, GAD1, GAD2	7
	BDNF/TrkB pathway	BDNF, NTRK2, NGF, NTRK1, NTF3, NTRK3, NTF4	7
	mTOR pathway	MTOR, RPTOR, RICTOR, MLST8, AKT1, AKT2, AKT3, PIK3CA, PIK3CB, PIK3CD, PIK3CG, PTEN, TSC1, TSC2, RHEB, RPS6KB1, RPS6KB2, EIF4EBP1, EIF4E	19
	CREB pathway	CREB1, CREB3, CREB5, ATF1, ATF2, ATF3, ATF4, CREBBP, EP300, CRTC1, CRTC2, CRTC3	12
	Immediate early genes	ARC, FOS, FOSB, FOSL1, FOSL2, JUN, JUNB, JUND, EGR1, EGR2, EGR3, EGR4, NPAS4	13
	CaMKII signaling	CAMK2A, CAMK2B, CAMK2D, CAMK2G, CAMK4, CALM1, CALM2, CALM3, CALML3, CALML4, CALML5, CALML6	12
	PKA/PKC signaling	PRKACA, PRKACB, PRKACG, PRKAR1A, PRKAR1B, PRKAR2A, PRKAR2B, PRKCA, PRKCB, PRKCG, PRKCD, PRKCE	12
	Sigma metabolic	CYP2B6, CYP2C19	2
5	Synaptic pruning modulators		
	Complement system	C1QA, C1QB, C1QC, C3, C4A, C4B, ITGAM, ITGB2, C3AR1, MASP1, MASP2, CFH, CFI, CFB, SERPING1	15
	Microglial function	CX3CR1, CX3CL1, TREM2, TYROBP, CD68, MERTK, AXL, TYRO3, GAS6, PROS1, MEGF10, GULP1, ABCA1, CD36, CD47, P2RY12, P2RY6, TMEM119	18
	Synaptic adhesion molecules	NRXN1, NRXN2, NRXN3, NLGN1, NLGN2, NLGN3, NLGN4X, CBLN1, CBLN2, CBLN4	10
	Semaphorin/Plexin	SEMA3A, SEMA3F, SEMA4D, SEMA6A, SEMA7A, PLXNA1, PLXNA2, PLXNA3, PLXNA4, PLXNB1, PLXNB2, NRP1, NRP2	13
	Ephrin signaling	EPHA4, EPHA7, EPHB1, EPHB2, EPHB3, EFNA1, EFNA2, EFNA3, EFNA4, EFNA5, EFNB1, EFNB2, EFNB3	13
	WNT signaling	WNT5A, WNT7A, WNT7B, FZD3, FZD5, RYK, LRP6, DVL1, GSK3B, CTNNB1, APC	11
	TGF-β/BMP signaling	TGFB1, TGFB2, TGFBR1, TGFBR2, BMP4, BMP7, BMPR1A, BMPR2, SMAD2, SMAD3, SMAD4	11
	Apoptotic pathways	CASP3, CASP6, CASP9, BAX, BAK1, BCL2, BCL2L1, APAF1, XIAP, DFNA5	10
	Ubiquitin-proteasome	UBE3A, FBXO45, MIB1, SMURF1, SMURF2, NEDD4, TRIM3, MYCBP2, USP14	9
	Cytoskeletal dynamics	RHOA, RAC1, CDC42, ROCK1, ROCK2, LIMK1, CFL1, CFL2, PAK1, ARPC2, WASF1, DAAM1	12
	Matrix metalloproteinases	MMP2, MMP9, MMP14, TIMP1, TIMP2, ADAM10, ADAM17, ADAMTS4	8
	Cell adhesion	NCAM1, L1CAM, CNTN1, CNTN2, CDH2, PCDH10, PCDHGA1, PCDHGB1, PCDHGC3	9
	Lipid metabolism	APOE, CLU, LDLR, LRP1, ABCA7, PLCG1, DGKZ	7
	MHC class I	B2M, HLA-A, HLA-B, HLA-C, TAP1, TAP2, PSMB8, PSMB9, CD3Z, LILRB3	10
	Ion channels	GABRA1, GABRB2, GABBR1, KCNJ10, SCN1A, CACNA1C, HCN1	7
	Activity-dependent regulators	SYNGAP1, FMR1, MECP2, LYNX1, OTX2, PVALB, SLC32A1, NPTX2, PENK	9
	Glial components	SPARC, SPARCL1, THBS1, THBS2, THBS4, GFAP, AQP4, GJA1	8
	Disease-associated genes	CSMD1, MIR137, ZNF804A, DISC1, ERBB4, NRG1, RELN, TCF4	8
	Additional regulators	CYFIP1, DSCAM, SDK1, SDK2	4
6	Autophagy and protein quality control		
	Core autophagy (MND)	C9orf72, SQSTM1, OPTN, TBK1, VCP, UBQLN2, CHMP2B, FIG4, ALS2, VAPB	10
	Protein aggregation and proteostasis	SOD1, TARDBP, FUS, ATXN2, ATXN3, ASAH1, LYST, SCP2	8
	Ubiquitin-proteasome system	UBA1, UBE1, UBQLN1	3
	Autophagy core machinery	ATG5, ATG7, ATG12, BECN1, ULK1, MAP1LC3A, MAP1LC3B, AMBRA1, RUBCN	9
7	RNA processing and metabolism		
	RNA-binding proteins and splicing	HNRNPA1, HNRNPA2B1, MATR3, EWSR1, TAF15, SETX, ANG	7
	SMN complex and snRNP	SMN1, SMN2	2
	Exosome complex and RNA degradation	EXOSC3, EXOSC8, EXOSC9	3
	mRNA export and translation	GLE1, IGHMBP2, RBM28	3
	Transcriptional regulators	VRK1, TRIP4, ASCC1, MORC2	4
	Stress granule components	G3BP1	1
	Riboflavin transport	SLC52A3, SLC52A2	2
8	Immune response and neuroinflammation		
	Innate immune response	TNIP1	1
	Glial and neuroinflammatory mediators	TREM2, TYROBP	2
	Complement system	C3, C1QA, C1QB, C1QC	4
	Cytokine signaling	IL1B, IL6, IL10, TNF, IFNG	5
	Inflammatory transcription	STAT3, RELA, NFKB1	3
	Toll-like receptors	TLR2, TLR4	2
	Inflammasome	NLRP3	1
	Receptor tyrosine kinase	ERBB3	1
